# Supplementary material for: Adsorption-induced slip inhibition for polymer melts on ideal substrates
Source: Nat Commun. 2018 Mar 21;9:1172. doi: 10.1038/s41467-018-03610-4 (PMC5862909; doi:10.1038/s41467-018-03610-4)
Supplement: Supplementary file 1 — Supplementary Information(PDF 176 kb) [file 41467_2018_3610_MOESM1_ESM.pdf]

Supplementary Information

"Adsorption-Induced Slip Inhibition for Polymer Melts on Ideal Substrates"

by Ilton et al.

Supplementary Note 1

Comparison of residence times for polymer molecules under flow

We wish to make a comparison of the expected residence times of adsorbed polymers between dewetting and levelling experiments. For this we want to compute

$$\tau_{\text{res}} = \tau_0 \exp\left(\frac{U_{\text{ads}} - F R_{\text{g}}}{kT}\right), \qquad (1)$$

where U_{ads} is the adsorption energy of a polymer chain on the substrate in the absence of an external force, F, and R_{g} is the polymer radius of gyration; we also make use of the thermal energy kT and the molecular relaxation timescale τ_0.

In hydrodynamic flow we take the force to be proportional to the pressure gradient, $\partial_x P$. Directly using the flow profiles shown in Supplementary Figure 1 (left axis) we can compute the Laplace pressure as

$$P = -\gamma\kappa = -\gamma \frac{\partial_x^2 h}{(1+(\partial_x h)^2)^{\frac{2}{3}}}, \qquad (2)$$

where we take the full expression for the pressure since the slopes of the contact line of the dewetting front are rather high (contact angle $\theta > 60°$). The right axes of Supplementary Figure 1 show the pressures thus calculated demonstrating an order of magnitude difference in the typical pressures in the region of flow. Computing the typical pressure gradient as $\partial_x P \approx \Delta P/\Delta x$,

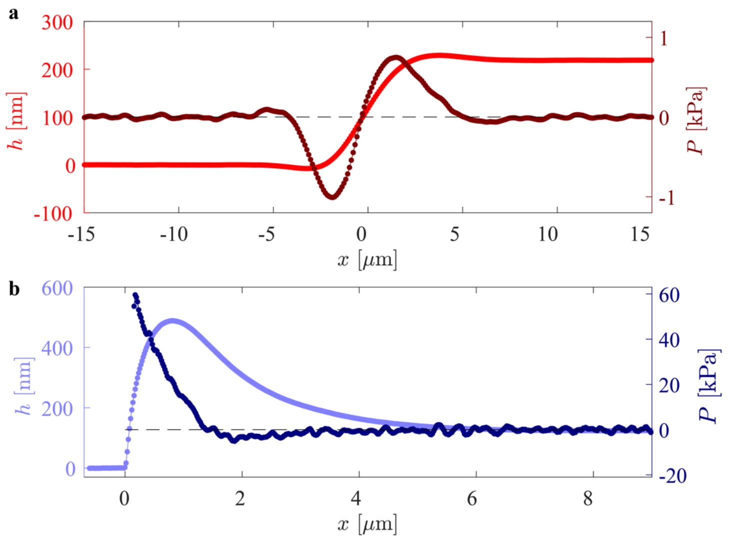

Supplementary Figure 1: Comparison of typical Laplace pressure in leveling and dewetting experiments. **a** A levelling stepped film composed of PS(192k) at T = 140°C after 300 min; the left axis shows the height profile $h(x)$ and the right axis gives the Laplace pressure computed according to Supplementary Equation 2. **b** A dewetting film composed of PS(186k) at T = 150°C with contact line radius R = 12 μm; the left and right axes are similar to those in the **a**.

where we take the difference between maximum and minimum pressures, we have

$$\partial_x P_{\mathrm{lev}} \approx 0.52\ \mathrm{kPa}\ \mu\mathrm{m}^{-1}\ , \qquad (3)$$

$$\partial_x P_{\mathrm{dew}} \approx 35\ \mathrm{kPa}\ \mu\mathrm{m}^{-1}\ . \qquad (4)$$

The net force on a molecule can be approximated by the pressure gradient multiplied by molecular volume $R_g{}^3$, therefore the typical added energy scale associated with an external force F in Supplementary Equation 1 is $FR_g \approx \partial_x P R_g^4$. For polystyrene of molecular mass 190 kg/mol, we have $R_g \approx 29$ nm [Fetters *et al.* Macromolecules, 1994, Table 1], we thus have

leveling: $FR_g \approx \partial_x P R_g^4 \approx 3.5 \times 10^{-22}\ \mathrm{J}\ ,$ (5)

dewetting: $FR_g \approx \partial_x P R_g^4 \approx 2.4 \times 10^{-20}\ \mathrm{J}\ .$ (6)

The typical thermal energy at 150°C, $kT \approx 5\times10^{-21}$ J, is also the expected energy scale of adsorption U_{ads}.

Our dewetting and levelling experiments cross the threshold across which we expect to find, respectively, no adsorption and significant adsorption.
